# A Rare Case of Salmonella Osteomyelitis in Immunocompetent Toddler Without Risk Factors

**DOI:** 10.7759/cureus.30642

**Published:** 2022-10-24

**Authors:** Alexandria E Daggett, Aaron L Heston, Mariel B Anderson, Leeann M Qubain, Angela F Veesenmeyer

**Affiliations:** 1 Department of Child Health, University of Arizona College of Medicine, Phoenix, USA; 2 Department of Pediatrics, Phoenix Children’s Hospital, Phoenix, USA; 3 Department of Pediatric Infectious Disease, Valleywise Health Medical Center, Phoenix, USA

**Keywords:** immunocompetent children, disseminated salmonella, salmonella osteomyelitis, epidural abscess, salmonella enteritidis

## Abstract

Salmonella infections are common, though rarely cause disseminated or severe disease in immunocompetent children. We present a case of severe salmonella osteomyelitis and epidural abscess in a patient without significant risk factors. This patient presented over the course of multiple visits with nonspecific symptoms of fever, malaise, and eventual joint pain. As symptoms progressed, the workup was broadened to find the eventual source of infection.

## Introduction

*Salmonella* species are among the most common causes of food-borne bacterial gastroenteritis, with the highest incidence in children under five years [1]. In most cases, these are self-limited infections. It is only recommended to treat salmonella infections with antibiotics if there are risk factors for invasive disease [2]. Despite the high incidence of salmonella infections, they are very infrequently associated with severe disease or sequelae in children. In children, the highest risk groups for invasive disease with salmonella are infants who have naturally immature immune systems and children with hemoglobinopathies or some form of immunocompromise [1]. We present a unique case of invasive and severe infection with salmonella leading to osteomyelitis and epidural abscess formation in a young, healthy, apparently immunocompetent toddler without known risk factors.

## Case presentation

A fully vaccinated 16-month-old Hispanic male with no previous medical history presented to an emergency department (ED) in Florida for tactile fever, vomiting, and diarrhea while away on vacation. A focal source of fever was not identified, and the parents were advised to continue supportive care. Three days later, the patient was seen by their primary care physician (PCP) via telemedicine at which time the symptoms had resolved.

Eleven days later, he presented to an Arizona emergency department (ED) with complaints of fever, decreased oral intake, and decreased urine output where a more thorough workup was obtained. Routine infectious and inflammatory laboratory parameters were within normal limits except for mildly elevated erythrocyte sedimentation rate (ESR) at 15.0 mm/hour and C-reactive protein (CRP) at 36.3 mg/L. Notably, he had normal white blood cells (WBC) and hemoglobin (Hgb) (Table 1). Cell count differential showed 34% segmented neutrophils, 1% bands, 55% lymphocytes, 4% monocytes, 1% eosinophils, and 0% basophils.

**Table 1:** Pertinent laboratory values throughout disease course. ED: emergency department; WBC: white blood cell; CRP: C-reactive protein; ESR: erythrocyte sedimentation rate

	Home ED presentation (14 days after symptom onset)	Pediatric infectious disease office (34 days after symptom onset)	Day of admission (36 days after symptom onset)
Laboratory values			
WBC (normal: 6.0-17.0×10^3^/µL)	15.0	17.1	17.8
CRP (normal: ≤5.0 mg/L)	36.3	9.8	28.7
ESR (normal: 0-10 mm/hour)	13	43	54
Platelets (normal: 150-600×10^3^/µL)	388	701	602

Over the next three weeks, the patient presented five times to his PCP’s office with similar complaints and no focal findings, at which point he was referred to the pediatric infectious disease (ID) clinic for a second opinion. Physical examination was normal, and laboratory results at this visit were again equivocal, notable only for the same mildly elevated ESR and CRP and now with mild leukocytosis (Table 1). A titer for coccidioidomycosis antibodies was drawn given the high prevalence in Arizona and was negative. An extensive exposure, travel, and family history were negative for any exposure or risk factors for salmonella, including lack of exposure to pets, reptiles, or other animals; history of frequent infection; family history of hemoglobinopathies; immunodeficiency; HIV infection; or other causes suspicious for salmonella infection. The only potential source identified was the 1-2 days of diarrhea in Florida, concerning for possible food-borne infection, despite no reported exposure to raw poultry, eggs, or known food outbreaks. Two days later, his symptoms worsened, the patient developed a new fever, and his symptoms now included difficulty walking and irritability with hip flexion while being held. This symptom progression resulted in admission to the hospital for a more thorough workup, now 36 days from the original ED visit.

**Figure 1:**
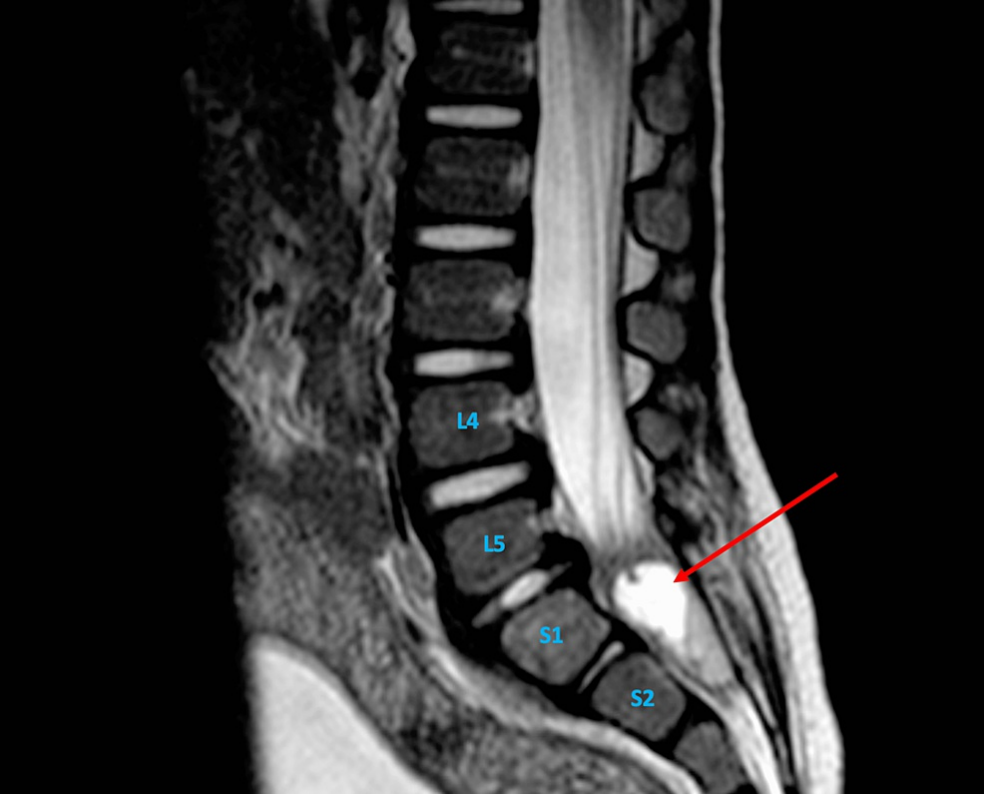
Sagittal MRI of the lumbar spine showing a contrast-enhancing region consistent with fluid collection that measures 2.3×0.9×1.3 cm in size (red arrow) and lies within the epidural space at the level of S1 and S2 vertebrae.

**Figure 2:**
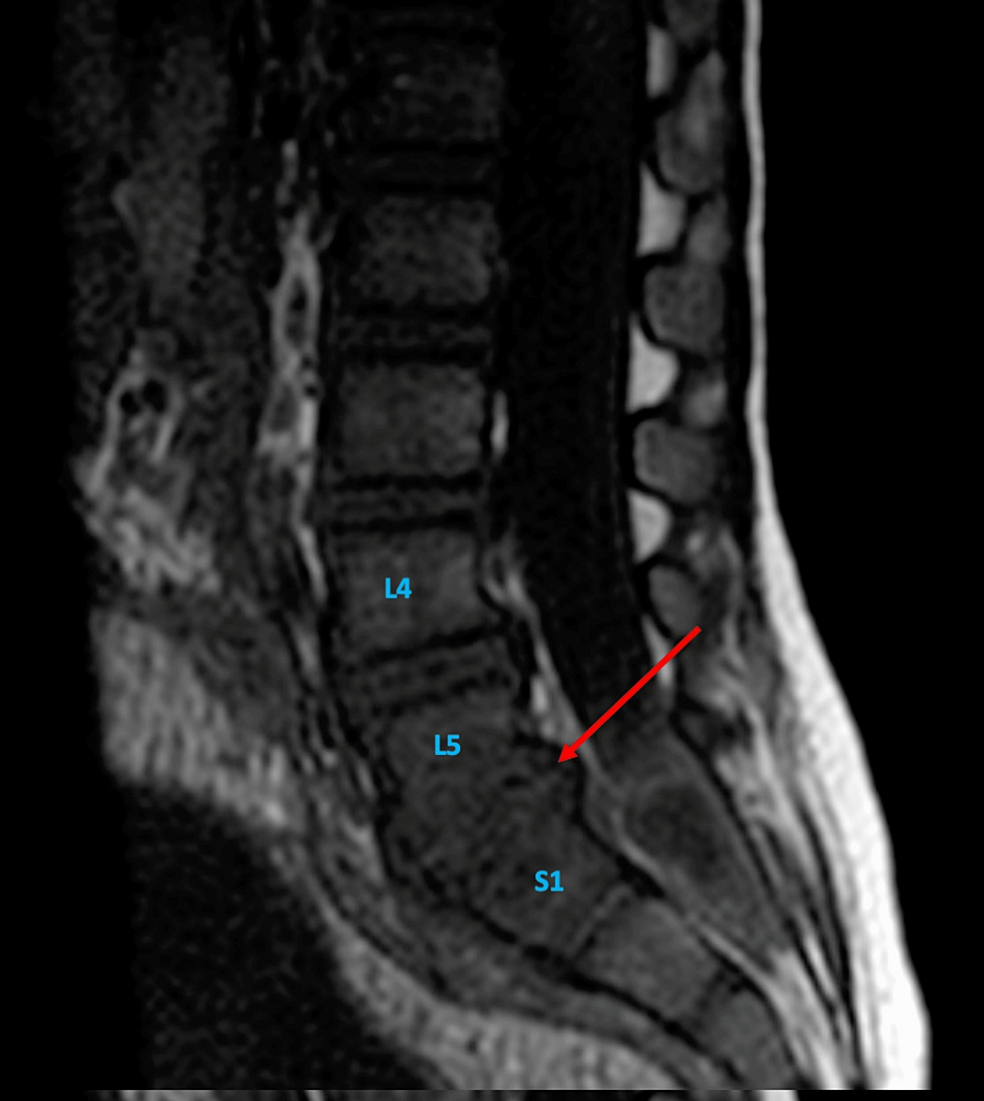
Sagittal T1-weighted MRI of the lumbar spine showing a hypointense marrow signal at L5 and S1 vertebral levels (red arrow).

**Figure 3:**
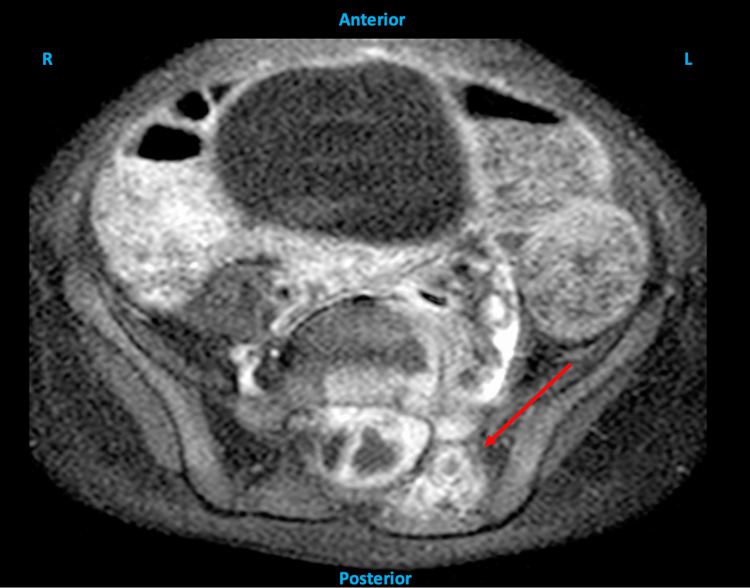
Axial MRI of the lumbar spine showing contrast enhancement in the dorsal paraspinal soft tissues surrounding the left L5-S1 facet joint (red arrow).

**Figure 4:**
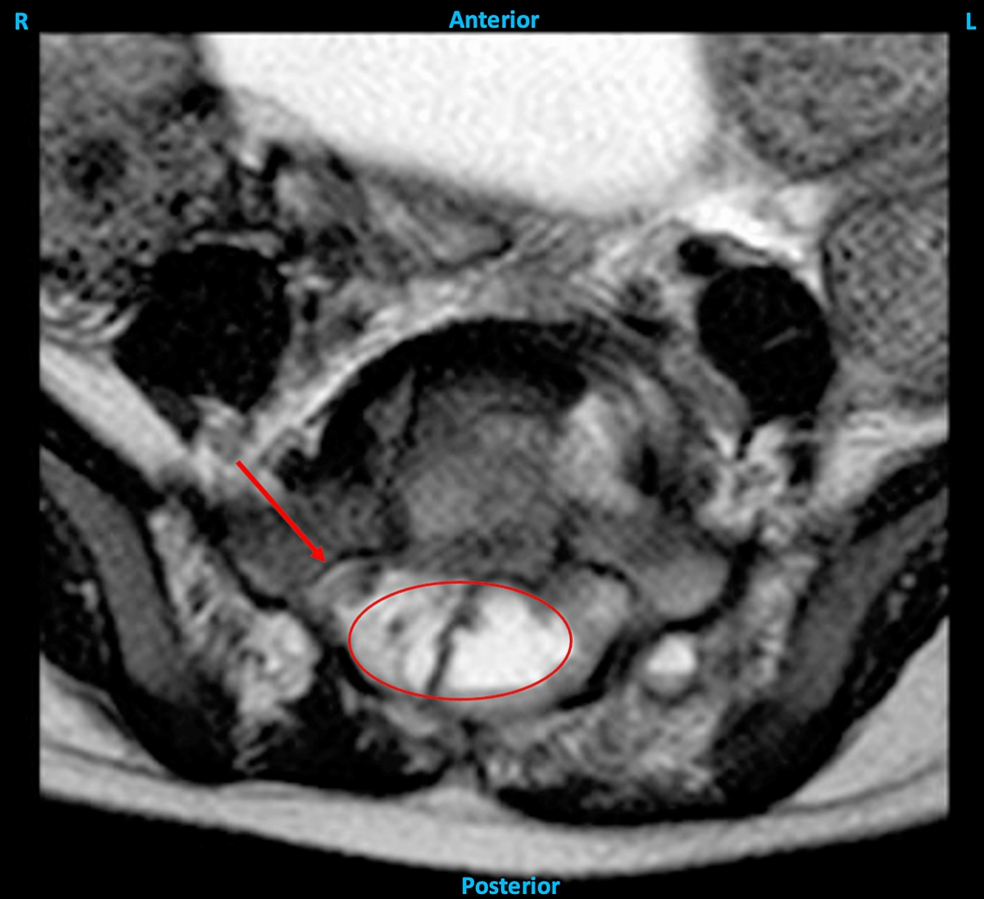
Axial MRI of the pelvis showing evidence of mass effect on the thecal sac (red circle) with rightward deviation of the sacral nerve roots (red arrow).

On admission, the patient was tachycardic with fever and exquisite tenderness to palpation of bilateral hips. There was a notable absence of pallor, lymphadenopathy, hepatosplenomegaly, or evidence of malnutrition, and the rest of his examination was normal. Blood and urine cultures were drawn. A peripheral blood smear (PBS) was obtained and showed normal RBC morphology with no evidence of hemoglobinopathy or sickled cells in particular. A hip ultrasound showed a trace effusion in the left hip, raising concern for possible septic joint, though the fluid collection was too small to aspirate. An MRI of the pelvis was done and showed mass effect on the adjacent thecal sac with rightward deviation of the sacral nerve roots. The MRI was expanded to encompass the lumbar spine and showed a peripherally contrast-enhancing fluid collection within the left dorsolateral aspect of the epidural space at the level of S1 and S2 measuring 2.3×0.9×1.3 cm. The associated contrast enhancement extended into the dorsal paraspinal soft tissues adjacent to the left L5-S1 facet. There was abnormal T1 hypointense marrow signal noted, most prominent at L5 and S1. These imaging results were suggestive of spinal epidural abscess and sacral osteomyelitis. The patient was transferred to the pediatric intensive care unit and prepared for emergency surgery (Figures 1-4).

Emergency left-sided laminectomy of L5, S1, and S2 was completed by neurosurgery the same evening. Intraoperatively, the abscess was densely adhered to the dura with full erosion into the distal thecal sac in the sacrum at S1 and S2. There was a pathologic spinal fluid leak that required repair with suture and onlay. Surgical wound cultures were positive for *Salmonella* species sensitive to ceftriaxone and ampicillin in the hospital laboratory. Cultures were subsequently sent to the state laboratory due to mandatory reporting and serotyping, which resulted as *Salmonella *Enteritidis IV. His blood and urine cultures on admission had no growth, and repeat blood cultures at the time of surgery remained negative. Postoperatively, the patient did well and was walking again prior to discharge from the hospital. The patient was treated with a six-week course of IV ceftriaxone followed by three months of oral amoxicillin. He was followed at regular intervals as an outpatient, and laboratory and physical examinations gradually returned to baseline.

## Discussion

Salmonella is a gram-negative bacteria that causes more than 1.3 billion cases of disease annually. Common sources of salmonella infection include raw poultry, contact with farm animals or animal feces, contact with reptiles, eggs, contaminated produce, international travel, and in some cases contaminated baby formula [1,3]. Salmonella is broadly classified into typhoidal and nontyphoidal salmonella, but there are nearly 2,500 classified serovars [4]. The subspecies *Salmonella *Typhi and *Salmonella *ParatyphiA, B, and C are known to cause typhoid fever (also known as enteric fever), with common symptoms including abdominal distress, nausea, vomiting, and diarrhea, which may progress to dysentery, fever, and flu-like symptoms. Nontyphoidal salmonella includes serovars such as *Salmonella *Typhimurium, *Salmonella *Choleraesuis, and *Salmonella *Enteritidis, many of which cause mild gastroenteritis, which resolves without antibiotics or other interventions. Additional presentations of invasive salmonella infection include bacteremia, meningitis, septic arthritis, and pneumonia. Osteomyelitis makes up only 0.8% of salmonella infections [5].

In addition to the above exposure risk factors, there are multiple well-documented patient risk factors for salmonella infection. Patients with sickle cell disease, hemoglobinopathies, and asplenia and patients who are immunocompromised are at higher risk for invasive salmonella infection. Patients with sickle cell disease are 200 times more likely to develop salmonella osteomyelitis than patients without [6].

This case represents a rare report of multifocal, vertebral osteomyelitis caused by nontyphoidal *Salmonella *Enteritidis in an apparently immunocompetent toddler without exposure risk factors. A review of the literature demonstrates very few documented cases of salmonella osteomyelitis in toddlers without sickle cell disease or other known risk factors. In one report, a two-year-old presented with vertebral osteomyelitis due to *Salmonella *Enteritidis, but this patient had sickle cell disease [6]. Another report describes a 17-month-old with nontyphoidal salmonella who had three days of diarrheal illness and recent head trauma followed by extradural abscess formation, but our patient did not have any notable trauma to increase the risk for abscess and osteomyelitis [7]. Other reports have described central nervous system salmonella abscess in a 12-month-old and 26-month-old following neurosurgical procedures [8,9]. There are various reports of salmonella osteomyelitis in teenagers and older children, who tend to have far more of the typical exposure and individual risk factors than our patient [5,10-17].

A recent institutional review from New Mexico revealed that 2% of osteomyelitis cases in immunocompetent pediatric patients were caused by nontyphoidal salmonella between 2003 and 2015. Of these, 75% had confirmed exposure to reptiles. These authors conducted a further literature review to create a theoretical case series of 42 cases, which demonstrated that lumbar bones were involved in 9/42 (21%) of cases. Multifocal osteomyelitis was uncommon, with 5/42 (12%) of cases [18]. Others report that 0.45%-2% of osteomyelitis in pediatric patients is caused by *Salmonella* species [5,10].

This patient’s cultured organism from surgical specimen was serotyped as *Salmonella *EnteritidisIV, which is among the top 100 most commonly isolated serotypes in the United States, though fairly uncommon in the patient’s home state, Arizona. Based on the Centers for Disease Control and Prevention surveillance reports on *Salmonella* species, *Salmonella *Enteritidis was the culture-confirmed cause of very few infections in Arizona and Florida, the state where he experienced a brief diarrheal illness, making up <0.1% and 8.4% of all salmonella infections, respectively [19]. In the absence of exposure to reptiles or farm animals or spinal trauma or instrumentation, it is likely that this patient had food-borne salmonella gastroenteritis as evidenced by a short course of loose stools while on vacation. This might have caused bacteremia, which seeded his osteomyelitis and resulting abscess formation. Such a sequence of events is exceedingly rare, as it is reported that nontyphoidal salmonella gastroenteritis in children progresses to transient bacteremia in only 1%-5% of pediatric patients, with only 3% of these immunocompetent patients (35% in immunocompromised) going on to develop severe consequences such as osteomyelitis, abscess, or meningitis [1]. Given the commonness of this serotype, the reason for the patient’s level of invasive disease remains unexplained.

## Conclusions

This case serves as an important reminder that close follow-up and repeated examinations are important in cases of undifferentiated illness. In this case, the differential was appropriately expanded as the physical examination changed, which ultimately led to definitive imaging not typically warranted by the initial presenting symptoms. This patient’s access to an integrated health system with regular follow-up and subspecialty care available is what ultimately led to successful intervention without long-term morbidity. It also emphasizes the need to maintain a broad differential diagnosis in young patients unable to vocalize or describe their symptoms and in whom it is more likely to have nonspecific and atypical symptoms of osteomyelitis.
